# Princeton Review

**Published:** 1878-09-20

**Authors:** 


### PRINCETON REVIEW.

This is an old and well established journal, being now in its 54th year. It deals with deep and important subjects mostly of a moral and religious bearing, written by able divines and profound thinkers. It is a large bi-monthly. Terms $2 per annum. Address Princeton Review, New York.

